# Supplementary material for: The Systems Biology Research Tool: evolvable open-source software
Source: BMC Syst Biol. 2008 Jun 29;2:55. doi: 10.1186/1752-0509-2-55 (PMC2446383; doi:10.1186/1752-0509-2-55)
Supplement: Additional file 1 — SBRT Archive. An archive of the current version of the Systems Biology Research Tool. [file 1752-0509-2-55-S1.zip › sbrt-1.4.0/doc/developers_guide/api/sbrt/shell/text/class-use/SimpleFormat.html]

Uses of Interface sbrt.shell.text.SimpleFormat


|  |  |  |
| --- | --- | --- |
| **Overview** \| **Package** \| **Class** \| **Use** \| **Tree** \| **Deprecated** \| **Index** \| **Help** | |  |
| PREV   NEXT | **FRAMES**    **NO FRAMES**     **All Classes** |  |


---


## **Uses of Interface sbrt.shell.text.SimpleFormat**

| Packages that use SimpleFormat | |
| --- | --- |
| **sbrt.shell.text** | Provides classes and interfaces for defining formats for basic objects. |
| **sbrt.shell.text.chem** | Provides classes and interfaces for defining formats for chemistry related objects. |
| **sbrt.shell.text.fba** | Provides classes and interfaces for defining formats for objects relevant to Flux Balance Analysis. |
| **sbrt.shell.text.math** | Provides classes and interfaces for defining formats for mathematical objects. |

Uses of SimpleFormat in sbrt.shell.text

| Subinterfaces of SimpleFormat in sbrt.shell.text | |
| --- | --- |
| `interface` | `BooleanFormat`             This interface is used to represent a format for boolean values. |
| `interface` | `CollectionFormat<E>`             This interface is used to represent collection formats. |
| `interface` | `DoubleFormat`             This interface is used to represent a format for double precision numbers. |
| `interface` | `IntegerFormat`             This interface is used to represent a format for integers. |
| `interface` | `LongFormat`             This interface is used to represent a format for longs. |
| `interface` | `PercentFormat`             This interface is used to represent a format for percentages. |

| Classes in sbrt.shell.text that implement SimpleFormat | |
| --- | --- |
| `class` | `BoolRelFormatV1<E>`             This interface is used to represent boolean relationship formats. |
| `class` | `DoubleFormatV1`             This class is a concrete implementation of `DoubleFormat`. |
| `class` | `DoubleFormatV2`             This class is a concrete implementation of `DoubleFormat`. |
| `class` | `FileFormatFormat`             This class is used to represent formats for types of file formats. |
| `class` | `IntegerFormatV1`             This class is a concrete implementation of `IntegerFormat`. |
| `class` | `LongFormatV1`             This class is a concrete implementation of `LongFormat`. |
| `class` | `NonEmptyStringFormat`             This class is used to define a format for `String` objects. |
| `class` | `PercentFormatVersion1`             This class is a concrete implementation of `PercentFormat`. |
| `class` | `SimpleListFormat<E>`             This interface is used to wrap `ListFormat` objects as `SimpleFormat` objects. |
| `class` | `SimpleSetFormat<E>`             This interface is used to wrap `ListFormat` objects as `SimpleFormat` objects. |
| `class` | `StringFormat`             This class is used to define a format for `String` objects. |
| `class` | `TrueFalseFormat`             This class is used to define a format for true/false values. |
| `class` | `YesNoFormat`             This class is used to define a format for yes/no values. |

| Methods in sbrt.shell.text that return SimpleFormat | |
| --- | --- |
| `SimpleFormat<E>` | `CollectionFormat.getElementFormat()`             Returns the format object used for the elements of this collection format. |
| `SimpleFormat<K>` | `EqualsMapEntryFormatV1.getKeyFormat()`             Returns the format used for keys. |
| `SimpleFormat<K>` | `ColonMapEntryFormatV1.getKeyFormat()`             Returns the format used for keys. |
| `SimpleFormat<V>` | `EqualsMapEntryFormatV1.getValueFormat()`             Returns the format used for values. |
| `SimpleFormat<V>` | `ColonMapEntryFormatV1.getValueFormat()`             Returns the format used for values. |

Constructors in sbrt.shell.text with parameters of type SimpleFormat

`AbstractLinkedHashSetFormat(java.lang.String delimiter,
java.util.regex.Pattern regex,
SimpleFormat<E> elementFormat)`
  
          Constructs a new `LinkedHashSet` format from
the provided arguments.

`AbstractListFormat(java.lang.String delimiter,
java.util.regex.Pattern regex,
SimpleFormat<E> elementFormat)`
  
          Constructs a new list format from the provided arguments.

`AmpersandSetFormat(SimpleFormat<E> elementFormat)`
  
          Constructs a new ampersand-delimited set format with the
provided element format.

`BoolRelFormatV1(SimpleFormat<E> elementFormat)`
  
          Constructs a new boolean relation format object that uses the
provided format for its elements.

`ColonMapEntryFormatV1(SimpleFormat<K> keyFormat,
SimpleFormat<V> valueFormat)`
  
          Constructs a new map entry format using the provided key and
value formats.

`ColonMapEntryFormatV1(SimpleFormat<K> keyFormat,
SimpleFormat<V> valueFormat)`
  
          Constructs a new map entry format using the provided key and
value formats.

`ColonMapFormatV1(SimpleFormat<K> keyFormat,
SimpleFormat<V> valueFormat)`
  
          Constructs a new map format using the provided key and
value formats.

`ColonMapFormatV1(SimpleFormat<K> keyFormat,
SimpleFormat<V> valueFormat)`
  
          Constructs a new map format using the provided key and
value formats.

`EqualsMapEntryFormatV1(SimpleFormat<K> keyFormat,
SimpleFormat<V> valueFormat)`
  
          Constructs a new map format using the provided key and
value formats.

`EqualsMapEntryFormatV1(SimpleFormat<K> keyFormat,
SimpleFormat<V> valueFormat)`
  
          Constructs a new map format using the provided key and
value formats.

`EqualsMapFormatV1(SimpleFormat<K> keyFormat,
SimpleFormat<V> valueFormat)`
  
          Constructs a new map format using the provided key and
value formats.

`EqualsMapFormatV1(SimpleFormat<K> keyFormat,
SimpleFormat<V> valueFormat)`
  
          Constructs a new map format using the provided key and
value formats.

`PipeListFormat(SimpleFormat<E> elementFormat)`
  
          Constructs a new pipe-delimited list format with the
provided element format.

`PipeSetFormat(SimpleFormat<E> elementFormat)`
  
          Constructs a new pipe-delimited set format with the
provided element format.

`TabListFormat(SimpleFormat<E> elementFormat)`
  
          Constructs a new tab-delimited list format with the
provided element format.

`TabSetFormat(SimpleFormat<E> elementFormat)`
  
          Constructs a new tab-delimited set format with the
provided element format.

`WhitespaceListFormat(SimpleFormat<E> elementFormat)`
  
          Constructs a new whitespace-delimited list format with the
provided element format.

`WhitespaceSetFormat(SimpleFormat<E> elementFormat)`
  
          Constructs a new whitespace-delimited set format with the
provided element format.

Uses of SimpleFormat in sbrt.shell.text.chem

| Subinterfaces of SimpleFormat in sbrt.shell.text.chem | |
| --- | --- |
| `interface` | `CatalystFormat`             This interface is used to represent the formats of catalysts in chemical reactions. |
| `interface` | `ChemSpeciesFormat`             This interface is used to represent the formats of chemical species. |
| `interface` | `RxnNameFormat<T>`             This interface is used to represent formats for the names of chemical reactions. |

| Classes in sbrt.shell.text.chem that implement SimpleFormat | |
| --- | --- |
| `class` | `CatalystFormatV1`             This class is a concrete implementation of `CatalystFormat`. |
| `class` | `CatalystFormatV2`             This class is a concrete implementation of `CatalystFormat`. |
| `class` | `ChemSpeciesFormatV1`             This class is a concrete implemenation of `ChemSpeciesFormat`. |
| `class` | `IrrevRxnFormatV1`             This class is used to format irreversible chemical reactions. |
| `class` | `IrrevRxnFormatV2`             This class is used to format irreversible chemical reactions. |
| `class` | `RevRxnFormatV1`             This class is used to format reversible chemical reactions. |
| `class` | `RevRxnFormatV2`             This class is used to format reversible chemical reactions. |
| `class` | `RxnNameFormatV1`             This class is a concrete implementation of `RxnNameFormat`. |

Uses of SimpleFormat in sbrt.shell.text.fba

| Subinterfaces of SimpleFormat in sbrt.shell.text.fba | |
| --- | --- |
| `interface` | `CatalystListFormat`             This interface is used to represent the formats of lists of catalyst names. |
| `interface` | `CatalystSetFormat`             This interface is used to represent the formats of sets of enzyme names. |
| `interface` | `RxnNameExprFormat<E extends MathExpr<S>,S>`             This interface is used to represent formats for mathematical expressions of reaction names. |
| `interface` | `RxnNameListFormat`             This interface is used to represent the formats of collections of reaction names. |
| `interface` | `RxnNameOrExprFormat`             This interface is used to represent formats for reaction names and mathematical expressions of reaction names. |
| `interface` | `RxnNameSetFormat`             This interface is used to represent the formats of collections of reaction names. |

| Classes in sbrt.shell.text.fba that implement SimpleFormat | |
| --- | --- |
| `class` | `CatalystListFormatV1`             This class is a concrete implemenation of `CatalystListFormat`. |
| `class` | `CatalystSetFormatV1`             This class is a concrete implemenation of `CatalystSetFormat`. |
| `class` | `CatalystVerifier`             This class is used to ensure catalysts exist in a given `CatalyzedFluxome`. |
| `class` | `ChemSpeciesVerifier`             This class is used to ensure chemical species exist in a given `Fluxome`. |
| `class` | `RxnNameExprFormatV1`             This class is used to format linear combinations of reaction names. |
| `class` | `RxnNameListFormatV1`             This class is a concrete implemenation of `RxnNameListFormat`. |
| `class` | `RxnNameOrExprFormatV1`             This class is a concrete implemenation of `RxnNameOrExprFormat`. |
| `class` | `RxnNameSetFormatV1`             This class is a concrete implemenation of `RxnNameSetFormat`. |
| `class` | `RxnNameVerifier`             This class is used to ensure reaction names exist in a given `Fluxome`. |

Uses of SimpleFormat in sbrt.shell.text.math

| Subinterfaces of SimpleFormat in sbrt.shell.text.math | |
| --- | --- |
| `interface` | `CoeffFormat`             This interface is used to represent the format of coefficients of mathematical expressions. |
| `interface` | `DirEdgeFormat<N>`             This interface is used to represent formats for the edges of directed graphs. |
| `interface` | `IndexPairFormat<R,C>`             This interface is used to represent formats for pairs of indices. |
| `interface` | `IntervalFormat`             This interface is used to represent formats for closed intervals. |
| `interface` | `LinearCombFormat<V>`             This interface is used to represent formats for linear combinations. |
| `interface` | `LinearCombTermFormat<V>`             This interface is used to represent the formats of terms from linear combinations. |
| `interface` | `MathExprFormat<E extends MathExpr<V>,V>`             This interface is used to represent formats for linear combinations. |
| `interface` | `MatrixElementFormat<R,C,E>`             This interface is used to represent formats for matrix elements. |

| Classes in sbrt.shell.text.math that implement SimpleFormat | |
| --- | --- |
| `class` | `BoundedIntervalFormatV1`             This class is a concrete implementation of `IntervalFormat`. |
| `class` | `CoeffFormatV1`             This class is a concrete implementation of `CoeffFormat`. |
| `class` | `CoeffFormatV2`             This class is a concrete implementation of `CoeffFormat`. |
| `class` | `DirEdgeFormatV1<N>`             This class is a concrete implementation of `DirEdgeFormat`. |
| `class` | `IndexPairFormatV1<R,C>`             This class is a concrete implementation of `IndexPairFormat`. |
| `class` | `IntervalFormatV1`             This class is a concrete implementation of `IntervalFormat`. |
| `class` | `LinCombTermFormatV1<V>`             This class is a concrete implementation of `LinearCombTermFormat`. |
| `class` | `LinCombTermFormatV2<V>`             This class is a concrete implementation of `LinearCombTermFormat`. |
| `class` | `LinearCombFormatV1<V>`             This class is a concrete implementation of `LinearCombFormat`. |
| `class` | `LinearCombFormatV2<V>`             This class is a concrete implementation of `LinearCombFormat`. |
| `class` | `MatrixElementFormatV1<R,C>`             This class is a concrete implementation of `MatrixElementFormat`. |
| `class` | `SenseFormat`             This class is used to represent formats of optimization senses. |

| Methods in sbrt.shell.text.math that return SimpleFormat | |
| --- | --- |
| `SimpleFormat<C>` | `IndexPairFormatV1.getColumnIndexFormat()`             Returns the format used for column indices. |
| `SimpleFormat<R>` | `IndexPairFormatV1.getRowIndexFormat()`             Returns the format used for row indices. |
| `SimpleFormat<V>` | `LinCombTermFormatV2.getVariableFormat()`             Returns the format used for variables. |
| `SimpleFormat<V>` | `LinCombTermFormatV1.getVariableFormat()`             Returns the format used for variables. |
| `SimpleFormat<V>` | `LinearCombFormatV2.getVariableFormat()`             Returns the format used for variables. |
| `SimpleFormat<V>` | `LinearCombFormatV1.getVariableFormat()`             Returns the format used for variables. |

Constructors in sbrt.shell.text.math with parameters of type SimpleFormat

`DirEdgeFormatV1(SimpleFormat<N> nodeFormat)`
  
          Constructs a new directed edge format.

`IndexPairFormatV1(SimpleFormat<R> rowIndexFormat,
SimpleFormat<C> columnIndexFormat)`
  
          Constructs a new index pair format.

`IndexPairFormatV1(SimpleFormat<R> rowIndexFormat,
SimpleFormat<C> columnIndexFormat)`
  
          Constructs a new index pair format.

`LinCombTermFormatV1(SimpleFormat<V> variableFormat)`
  
          Constructs a new linear combination term format using
the provided formats.

`LinCombTermFormatV2(SimpleFormat<V> variableFormat)`
  
          Constructs a new linear combination term format using
the provided formats.

`LinearCombFormatV1(SimpleFormat<V> variableFormat)`
  
          Constructs a new linear combination format using the provided format.

`LinearCombFormatV1(SimpleFormat<V> variableFormat,
double zeroCutoff)`
  
          Constructs a new linear combination format using the provided arguments.

`LinearCombFormatV2(SimpleFormat<V> variableFormat)`
  
          Constructs a new linear combination format using the provided format.

`LinearCombFormatV2(SimpleFormat<V> variableFormat,
double zeroCutoff)`
  
          Constructs a new linear combination format using the provided arguments.

`MatrixElementFormatV1(SimpleFormat<R> rowIndexFormat,
SimpleFormat<C> columnIndexFormat)`
  
          Constructs a new matrix element format.

`MatrixElementFormatV1(SimpleFormat<R> rowIndexFormat,
SimpleFormat<C> columnIndexFormat)`
  
          Constructs a new matrix element format.

---


|  |  |  |
| --- | --- | --- |
| **Overview** \| **Package** \| **Class** \| **Use** \| **Tree** \| **Deprecated** \| **Index** \| **Help** | |  |
| PREV   NEXT | **FRAMES**    **NO FRAMES**     **All Classes** |  |


---
